# Health service utilization patterns of primary care patients with osteoarthritis

**DOI:** 10.1186/1472-6963-7-169

**Published:** 2007-10-23

**Authors:** Thomas Rosemann, Stefanie Joos, Joachim Szecsenyi, Gunter Laux, Michel Wensing

**Affiliations:** 1 University Hospital Heidelberg, Department of General Practice and Health Services Research; Vosstr. 2, 69115 Heidelberg, Germany; 2 Radboud University Nijmegen Medical Centre, 6500 HB Nijmegen, The Netherlands

## Abstract

**Background:**

To assess factors associated with visits to GPs, orthopaedists, and non-physician practitioners of complementary medicine (alternative practitioners) by primary care patients with osteoarthritis (OA).

**Methods:**

Cross-sectional survey among 1250 consecutively addressed patients from 75 primary care practices in Germany. All patients suffered from OA of the knee or hip according to ACR criteria. They received questionnaires collecting sociodemographic data, data about health service utilisation, prescriptions, comorbidities. They also included established instruments as the Arthritis Impact Measurement Scale (AIMS2-SF) to assess disease-specific quality of life and the Patient Health Questionnaire (PHQ-9) to assess depression. Hierarchical stepwise multiple linear regression models were used to reveal significant factors influencing health service utilization.

**Results:**

1021 of 1250 (81.6%) questionnaires were returned. Nonrespondents did not differ from participants. Factors associated with health service use (HSU) varied between providers of care. Not being in a partnership, achieving a high score on the PHQ-9, increased pain severity reflected in the “symptom” scale of the AIMS2-SF, and an increased number of drug prescriptions predicted a high frequency of GP visits. The PHQ-9 score was also a predictor for visits to orthopaedists, as were previous GP contacts, a high score in the "symptom" scale as well as a high score in the "lower limb scale" of the AIMS2-SF. Regarding visits to alternative practitioners, a high score in the AIMS -"social" scale was a positive predictor as older people were less likely to visit them.

**Conclusion:**

Our results emphasize the need for awareness of psychological factors contributing to the use of health care providers. Addressing the revealed factors associated with HSU appropriately may lead to decreased health care utilization. But further research is needed to assess how this can be done successfully.

## Background

Osteoarthritis (OA) substantially impacts health-related quality of life [1]. As a consequence, patients with OA frequently require access to a broad range of healthcare services [2,3]. Furthermore, since OA is highly prevalent among older people, the economic burden of OA is also immense [4-6]. Even though surgical interventions are a well-established evidence-based treatment option, patients with osteoarthritis which is sufficiently severe to consider joint replacement represent a minority in primary care [7]. Thus, in most cases, the GP is the main care provider for many OA patients over a long period and plays an important role regarding the HSU of these patients [3]. In Germany, the GP has some kind of gate-keeper role since patients who visit a specialist, e.g. the orthopaedist, without visiting the GP first have to pay an additional fee. In consequence most visits to orthopaedists are preceded by a GP consultation. Furthermore, according to official statistics of the Central Institute of the German Health Insurances (Zentralinstitut für die kassenärztliche Versorgung in der Bundesrepublik Deutschland), referrals to orthopaedists are the most frequent ones among all referrals from GPs to specialists. These statistics also show that referral rates to orthopaedists increased notably over the last years, even though GPs considered many of these referrals to be avoidable. It is quite obvious that inappropriate referrals increase costs without improving care. Knowledge of factors associated with HSU can therefore help to reduce costs [8]. Considering this, it is quite astonishing that regarding OA patients, little is known about factors associated with visits to GPs but also with contacts to orthopaedists. Hagglund et al. assessed health service utilization (HSU) in a sample of OA and rheumatoid arthritis RA patients [2]. In that study, the strongest predictor of health care utilization was the prior use of the system. The number of physician visits in the past, number of received prescriptions, and hospitalization probability were further important predictors. Cronan et al. revealed increased age, increased impairment, lower well-being scores, and additional comorbidities as predictors of health care use [9]. But when assessing HSU, most of prior studies did not distinguish between GP contacts and contacts to specialists. Furthermore, important comorbidities like depression were not assessed.

Since the GP is the main gateway to health care system for patients with OA, the aim of our study was to assess factors associated with the use of GPs and orthopaedists in a large sample of OA patients. Furthermore, since it is known that use of complementary and alternative medicine (CAM) is very common among patients, we assessed factors for visits to so-called "alternative practitioners" (non-physician state-registered practitioners of CAM), who are very popular among patients in Germany, but also in other health care systems worldwide [10-13].

## Methods

The data used for this study were retrieved from the PraxArt-project, which aimed to learn about the present care of OA patients in primary care and aimed to improve the QoL of patients by appropriate interventions later on [14]. The presented study aimed at describing health service utilization patterns of osteoarthritis patients. The project was financed over a period of 6 years by the German Ministry for Education and Research (BMBF) and comprised data from a sample of 75 representative general practitioners in the area of Baden-Wuerttemberg and Bavaria. The data used in this study were retrieved from the baseline assessment of the project and were collected between March and May 2005 as described below.

### Patient inclusion criteria

Patients were addressed consecutively according to their appearance in the GP practices. To be eligible for inclusion, patients had to be adult and diagnosed with osteoarthritis of the hip or the knee according to the ACR criteria [15,16]. To avoid the bias of overrepresentation of patients with a number of comorbidities, they were only asked to participate if the reason for the current encounter was related to OA. At baseline, neither the GP nor the patients were exposed to any intervention. The sample can be regarded as representative for OA patients in primary care in Germany.

### Data collection

After giving their written informed consent patients received the questionnaire and a return envelope with the postal address of the university. Each GP distributed questionnaires to 15 patients. The patients were asked to return the questionnaire by mail to the university. Neither the GP nor the practice team had any possibility to get knowledge of patients' answers. All collected data referred to a period of 6 months prior to the survey. Each questionnaire was linked with an identification number to the participants' list kept in the practices, so that data given by patients could be checked by comparing them with the patients' file.

Data about health service utilization were collected by asking for instance, "How often did you visit an orthopaedic specialist within the last 6 months?" As far as possible, patients' answers were checked by comparing them with the patients' files. Thus, reliability of patients' answers could be assessed later on in the project. If differences occurred, the data of the medical file were used. Since it is known that patients with depressive disorders are high utilizers [17,18] of the health care system and depression shows increased prevalence among patients with arthritis [19,20], we assessed depression by means of the PHQ-9. The PHQ-9 is a short form of the PHQ-D questionnaire, which has proven to be a valid instrument for those assessments [21,22]. The impact of OA on patients' QoL was assessed by the German AIMS2-SF [23,24]. This instrument is the most widespread tool to assess QoL of patients with arthritis. It provides a comprehensive assessment of patients' health status comprising the dimensions physical limitation (divided into upper body limitation and lower body limitation), symptom (reflecting perceived pain), social (reflecting social contacts), affect (reflecting mood), and work (reflecting the ability to work). Higher scores in the AIMS2-SF indicate lower QoL. The patient questionnaire additionally comprised information about sociodemographic data (sex, age, educational level, working situation, family situation) and the following diseases as comorbidities: High blood pressure (HBP), diabetes, heart insufficiency, coronary heart disease, elevated cholesterol level (> 200 mg/dl), ulcer or stomach disease, asthma/chronic obstructive pulmonary disease (COPD), kidney disease, cancer, and stroke. The data were transferred into the SPSS program (version 12.0). The study protocol was approved by the ethics committee of the University of Heidelberg prior to the start of the study in January 2005 (approval number 021/2005).

### Statistical analysis

Correlations between sociodemographic data, clinical variables and visits to the different health care providers were assessed by means of computing Pearson's r. If a linear relationship, which is required for computing Pearson's r, could not be confirmed by pre-testing with scatter plots, Spearman's rho was used instead. Scatter plots were also performed -where applicable- to confirm linear relationship and to enable the linear regression models. Only factors which showed significant correlations (p < 0.05) were selected to be entered in the regression model. To reveal factors associated with HSU, three linear regression models with "contacts to GPs", "contacts to orthopaedists", and "contacts to alternative practitioners"' as dependent variables were performed. A hierarchical stepwise technique with the sociodemographic variables entered in the first block and disease characteristics in a second block was used. This was necessary to avoid an artificially high *R*^2 ^due to forced entry in the model of highly correlated factors. This approach represents – in a statistical sense – as conservative [25].

## Results

In total 1311 patients were addressed by the GPs, 1250 of them agreed to receive the package of questionnaires; 1021 (77.9%) packages were returned to the university. From each practice at least 11 packages were returned. The main reason given for nonparticipation was time effort. In 271 questionnaires were data missing. In 123 cases data could be completed by retrieving them from the medical file. In no item of the AIMS2-SF and the PHQ-9 occurred more than 5 % missing data. The PHQ-9 scores could be calculated in 1012 cases. A comparison of the 1021 respondents to the nonrespondents revealed no significant differences regarding sociodemographic variables (age, gender), disease characteristics (duration of disease) as well as number of comorbidities, health service utilization (only contacts to GPs and orthopaedists could be checked) and prescribed medication.

Table 1 displays the characteristics of the study sample. Mean age was 66.1 years and the mean duration of OA was 13.7 years. 347 (34.0%) of the 1021 included patients were male and 674 (66.0%) were female. Completely retired from work were 233 (67.1%) men and 482 (71.5%) women. As a consequence of the high rate of retired persons in the study sample, the work scale of the AIMS2-SF was excluded from the regression analysis since it applied only to patients still working.

**Table 1:** Characteristics of the study sample (n = 1021)

		Mean/n	SD/%
Female		674	66.0%
Age		66.1	15.1
Duration of OA (years)		13.7	12.8
Body Mass Index (BMI)		28.2	4.9
Married/Living in partnership		654	64.1%
Years of education	>=9	720	71.9%
	10–13 years	156	15.6%
	> 13 years	125	12.5%
Visits in the last 6 months	GPs	5.1	7.9
	Orthopeadists	1.8	3.6
	Alternative healers	0.30	2.7
OA related prescriptions		1.7	0.8
No. of comorbid conditions (0–10)		2.2	1.7
	High blood pressure	565	55.3%
	Elevated cholesterol	369	36.1%
	Diabetes	177	17.3%
	Heart Insufficiency	194	19.0%
	Coronary vessel disease	132	12.9%
	Ulcer/Gastritis	223	21.8%
	Asthma/COPD	98	9.6%
	Renal Insufficiency	56	5.5%
	Cancer	37	3.6%
	Stroke	46	4.5%

The most common comorbidity was high blood pressure, followed by elevated cholesterol and a (history of) ulcer or severe gastritis. Table 1 also displays the frequencies of contacts to the different provider of health services with a mean of 5.1 contacts to GPs (SD 7.9), 1.8 (3.6) to orthopaedists and 0.3 (2.7) to alternative practitioners.

Table 2 shows the bivariate correlations between sociodemographic characteristics respectively disease characteristics and the different health care providers. Factors which achieved a significant correlation and which were consequently entered in the respective regression model are displayed in bold figures. Regarding contacts to GPs, none of the variables showed a notably high correlation. BMI and, interestingly, the social scale of the AIMS2-SF did not show significant correlation to the frequency of GP contacts. Consequently, these variables were not entered in the regression model. Regarding contacts to orthopaedists, the variables "previous contacts to GPs", "number of comorbidities" as well as "disease duration" were not entered in the regression model since they achieved no significant correlation.

**Table 2:** Correlations between patients' variables and visits to GPs, orthopaedists and alternative practitioners

	GP*	p	Orthopaedic*	p	Alternative practitioner*	p
Gender	**0.122**	**0.000**	**0.023**	**0.048**	0.001	0.982
Age	0.050	0.169	**-0.139**	**0.000**	**-0.095**	**0.012**
Marital status	**-0.073**	**0.030**	**0.065**	**0.049**	0.035	0.314
Previous GP contacts	0.082	0.234	**0.008**	**0.000**	0.009	0.303
Educational level	-0.054	0.112	**0.055**	**0.009**	-0.003	0.939
Previous visits to orthopaedists	0.008	0.234	**0.214**	**0.000**	-0.037	0.303
Disease duration	0.066	0.052	0.013	0.709	-0.034	0.331
Lower body	**0.195**	**0.000**	**0.173**	**0.000**	0.000	0.996
Upper body	**0.165**	**0.000**	0.026	0.439	0.033	0.345
Symptom	**0.282**	**0.000**	**0.254**	**0.000**	0.015	0.671
Affect	**0.187**	**0.000**	**0.155**	**0.000**	0.049	0.171
Social	0.026	0.447	0.001	0.983	**0.082**	**0.018**
Work	0.217	**0.002**	0.224	0.001	0.163	0.024
BMI	-0.006	0.848	0.024	0.481	**-0.073**	**0.037**
PHQ-9 sum score	**0.197**	**0.000**	**0.145**	**0.000**	0.061	0.106
Number of comorbidities	0.046	0.171	-0.001	0.978	0.000	0.988
Prescribed drugs	**0.256**	**0.003**	0.736	**0.019**	0.081	**0.010**

* Spearmen rho values; significant correlations in bold, bold variables were entered in the respective regression model

The small amount of variables achieving significant p-values for alternative practitioners already indicated that only a small number of factors may predict the choice of these kind of health care providers. Consequently, "gender", "marital status", educational level", "number of comorbidities", and "previous contacts to GPs" were not entered in the regression model. Also the scales of the AIMS2-SF indicating physical limitation were not entered since the values for the correlation analysis indicated already that there might be no relation of these variables with the contacts to alternative practitioners.

Table 3 displays the results of the regression model for GP contacts as dependent variable. With a beta of 1.429 (p = 0.001), not living in a partnership was the strongest factor associated with visits to the GP. Also, the amount of prescribed drugs was an important positive predictor (β = 0.641; P = 0.001) for GP consultations. Furthermore, perceived pain (β = 0.605; p = 0.014) and low mood or depression, reflected in a higher PHQ-9 score (β = 0.352; p < 0.0009) represented significant factors associated with visits to GPs in our study sample.

**Table 3:** Factors associated with GP contacts assessed by stepwise regression

Dependent: GP contacts Unadjusted R^2 ^= 0.359 Adjusted R^2 ^= 0.327 F = 15.346; p < 0.0001	Beta	SE	Change in R^2^	p
(constant)	1.516	1.216		0.213
Marital status (living alone)	1.439	0.495	0.170	0.001
Number of prescribed drugs	0.641	0.278	0.096	0.001
Symptom*	0.605	0.244	0.075	0.014
PHQ-9 score	0.352	0.085	0.018	0.000

* AIMS2-SF scale

As can be seen in table 4, the most important predictor of increased contacts to orthopaedists was a high score in the PHQ-9 (β = 0.509; p = 0.013). The beta for this factor was notably higher than for "GP visits". Having more pain and perceiving more physical limitation of the lower limb, which both indicate more severity of OA, were also positive factors associated with visits to orthopaedists. Finally the probability to visit orthopaedists increased with the amount of GP contacts, but with 0.035 the beta was quite low (p = 0.064).

**Table 4:** Factors associated with contacts to orthopaedists assessed by stepwise regression

Dependent: contacts to orthopaedists		beta	SE	Change in R^2^	P
Unadjusted R^2 ^= 0.399	(constant)	0.832	0.437		0.057
	PHQ-9-score	0.509	0.130	0.169	0.013
Adjusted R^2 ^= 0.369	Symptom*	0.445	0.125	0.151	0.001
F = 16.236 ; p < 0.001	Lower body*	0.241	0.120	0.056	0.048
	GP contacts	0.035	0.019	0.023	0.064

* AIMS2-SF scales

The analyses of correlations indicated already that only few factors associated with may exist regarding visits to alternative practitioners. The only significant (p = 0.024) positive predictor that remained in the regression analysis was the social scale of the AIMS2-SF. A high score in the social scale reflects few social contacts and little social support. The beta of 0.202 (p = 0.024) indicates that this predicts increased numbers of encounters with alternative practitioners (table 5). Age was a slightly negative predictor (β = -0.078; p = 0.012) indicating that increased age reduces the probability to visit alternative practitioners. The adjusted R^2 ^of 0.322 also indicates that this model explains a smaller amount of variation in the dependent variable as the other two models.

**Table 5:** Factors associated with contacts to alternative practitioners assessed by stepwise regression

Dependent: contacts to alternative practitioners Unadjusted R^2 ^= 0.411 Adjusted R^2 ^= 0.322 F = 17.214 ; p < 0.001	beta	SE	Change in R^2^	p
(constant)	0.402	0.212		0.045
Social	0.202	0.017	0.387	0.024
Age	-0.078	0.005	0.024	0.012

## Discussion

Factors associated with health service use varied between providers of care. In our study sample factors associated with GP visits were not being in a partnership, increased PHQ-9 scores, increased pain (reflected in the "symptom" score), and an increased amount of prescribed drugs. Suffering from increased physical limitation of the lower limb or pain intensity were associated with a higher probability to visit an orthopaedic specialist as well as a higher score on the PHQ-9 and increased previous contacts to GPs. Contrary to this, only two factors associated with visiting an alternative practitioner could be revealed: few social contacts, reflected in the social scale of the AIMS2-SF, and younger age. Increased age reduced the probability to visit an alternative practitioner slightly.

The revealed factors associated with GP visits seem plausible: It is known that patients with low mood or depression are high utilizers of the health care system [17,18,26]. Nevertheless, prior studies with arthritis patients did not assess depression as a possible factor for HSU. Prior studies also indicated that physicians sometimes tend to ignore depression and consider more physical factors when estimating QoL of patients with osteoarthritis [27,28]. Furthermore, as in many chronic diseases, prevalence of depression is increased among OA patients [19]. So, our findings emphasize the importance to be aware of depression not only with respect to QoL but also regarding HSU. Another important factor for HSU in our study was pain, reflected in the AIMS2-SF "symptom"-scale. Since pain constitutes one of the major burdens of the disease this result is not surprising and has already been shown for instance by Dominick et al [29-31]. In their study, pain was the only predictor that remained in the multivariate regression model [7]. Another factor remaining in our regression model was the "social" scale, reflecting patients' social situation and social support. Regarding social support, Cronan et al. showed that social factors contribute to HSU-patterns of arthritis patients and that increasing social support can reduce HSU [30]. An important reason for that is most probably the association between social support and pain [32]. This is emphasized by our result that living in a partnership was associated with fewer HSU. The importance of support provided by the spouse has been shown in a study by Keefe et al., where spouse assistance increased the effect of interventions to improve QoL of OA patients [33]. The most modest factor associated with GP visits was the number of prescribed drugs. It is quite obvious that this results in frequent GP contacts; this was already found in previous studies [7]. But interestingly, the number of prescribed drugs and not the quantity of comorbidities remained as a predictor in the final model.

Physical limitation and perceived pain represent the major burden of disease and might also represent the biggest challenge for the treating GP [34]. In this context, their remaining in the model as important factors associated with is not surprising but rather in concordance with previous findings. Physical limitation has been shown before to be an important predictor for HSU [9]. Interestingly, a high score on the PHQ-9, reflecting low mood or a real depressive disorder was a predictor for GP visits as well as for visits to the orthopaedist. It seems that these patients do not only visit their GP more often, they were also referred more often. It remains unclear whether the reason for the referrals is the GPs' intention to get rid of perceived pressure or if severity of OA within this patient group is overestimated compared to nondepressed patients.

In Germany, CAM becomes more and more important to patients: Data show that the overall percentage of individuals who experienced CAM increased from 52% in 1970 to 73% in 2002 [12]. The most frequently used CAM among German patients are herbal medicine, exercise therapy and hydrotherapy [36]. Among arthritis patients, the use of CAM is also quite popular and alternative practitioners are important providers of these treatments [12,37,38]. Interestingly, less is known about patients' motives to do so and physicians tend to ignore this phenomenon [13,39]. Our results are in accordance with a previous survey in Germany, showing that younger and better educated patients tend more to CAM [36]. What our study adds regarding CAM is that social factors are also of great importance since less social support was associated with a higher probability to visit practitioners of complementary medicine. This may reflect the desire for a treatment including more attention, empathy or simply more time with a care provider [30]. The German health care system provides unlimited access to GPs as well as to specialists, and the health insurances nearly cover all arising costs of GP and specialists' care. Accordingly, instrumental diagnostics and treatments are predominant in this system, but physicians often lack the time for talking. In Germany only a small part of CAM is covered by the statutory health insurance (SHI), but only if provided by a physician. In detail, only chiropractic, classic naturopathy to some extent and, in some cases, acupuncture, is covered by the SHI. All remaining CAM methods are not covered by SHI but have to be paid by the patients themselves. Costs of alternative practitioners-regardless what kind of CAM is provided – are generally not covered by the SHI. The fact that patients are willing to pay for such a treatment out-of-pocket should increase physicians' awareness for this dimension of QoL of chronically ill patients. Interestingly, older people seem to tend a little bit less to visit alternative practitioners than younger ones. Various reasons may be responsible for this finding; one could be that older ones are frequently more satisfied with received care by their GP than younger ones. Financial reasons may also contribute to this finding since CAM has to be paid by the patients themselves which may constitute a barrier to CAM especially for older patients. Further research would be necessary to explore patients' opinion in this context. Although these findings are characteristic for the German health care system, they may be transferred to other systems, where the use of CAM also plays an increasing role in health care.

### Strength and Weaknesses

Some weaknesses of our study have to be acknowledged: First of all, the data are retrieved in a cross-sectional study and the revealed factors influencing HSU patterns have to be regarded more as "associated factors" than as "predictors". Secondly, since the data were collected in primary care patients, they can not be easily transferred to the whole population. Patients who directly visit the orthopaedist or the alternative practitioner were missed in our survey. But in this context it has to be noticed that this may represent only a small minority since most patients aim to avoid the additional fee which occurs when they visit the specialist without consulting the GP first. Altogether the bias related to the fact that the data were collected in the GP practices can be assumed as small. Regarding the amount of prescriptions, which was also revealed as an important factor for GP contacts, it may be difficult to distinguish if this is the effect of many GP contacts or rather a predictor. Regarding the data about CAM some further limitations should be noticed: Data on contacts to alternative practitioners were self-reported and may be of lower validity than data about contacts to GPs or orthopaedists which where retrieved from patients' files. Furthermore, it remains unclear if and to what extent our findings can be transferred to other samples or with other diseases. On the other hand, this study enrolled not only a large number of primary care patients; it is also the first study assessing factors associated with the use of different health care providers separately. Furthermore, it controlled important factors such as depression which have been ignored in prior studies.

## Conclusion

Even though factors vary between the different providers of care the contribution of social factors as well as psychological factors such as depression is enormous and might be underestimated. The need of physicians' awareness of these factors is emphasized. The finding that (younger) patients are even willing to pay for empathy and time as provided by alternative practitioners emphasizes patients' desire for being regarded holistically. Physicians should always be aware that the target is the whole patient and not only the joint.

## Competing interests

The author(s) declare that they have no competing interests.

## Authors' contributions

TR and SJ conceived and performed the study and drafted the manuscript. GL and JS performed the data management and statistical calculations. MW participated in the study design and revision of the manuscript. All authors read and approved the final manuscript.

## Pre-publication history

The pre-publication history for this paper can be accessed here:



## References

[B1] Lam CL, Lauder IJ (2000). The impact of chronic diseases on the health-related quality of life (HRQOL) of Chinese patients in primary care. Fam Pract.

[B2] Hagglund KJ, Clark MJ, Hilton SA, Hewett JE (2005). Access to healthcare services among persons with osteoarthritis and rheumatoid arthritis. Am J Phys Med Rehabil.

[B3] Peat G, McCarney R, Croft P (2001). Knee pain and osteoarthritis in older adults: a review of community burden and current use of primary health care. Ann Rheum Dis.

[B4] Woolf AD, Pfleger B (2003). Burden of major musculoskeletal conditions. Bull World Health Organ.

[B5] March LM, Bachmeier CJ (1997). Economics of osteoarthritis: a global perspective. Baillieres Clin Rheumatol.

[B6] Leardini G, Salaffi F, Caporali R, Canesi B, Rovati L, Montanelli R (2004). Direct and indirect costs of osteoarthritis of the knee. Clin Exp Rheumatol.

[B7] Dominick KL, Ahern FM, Gold CH, Heller DA (2004). Health-related quality of life and health service use among older adults with osteoarthritis. Arthritis Rheum.

[B8] Groessl EJ, Cronan TA (2000). A cost analysis of self-management programs for people with chronic illness. Am J Community Psychol.

[B9] Cronan TA, Shaw WS, Gallagher RA, Weisman M (1995). Predicting health care use among older osteoarthritis patients in an HMO. Arthritis Care Res.

[B10] Dieppe P, Brandt KD (2003). What is important in treating osteoarthritis? Whom should we treat and how should we treat them?. Rheum Dis Clin North Am.

[B11] Rao JK, Mihaliak K, Kroenke K, Bradley J, Tierney WM, Weinberger M (1999). Use of complementary therapies for arthritis among patients of rheumatologists. Ann Intern Med.

[B12] Marstedt G MS (2002). Gesundheitsberichterstattung des Bundes: Inanspruchnahme alternativer Methoden in der Medizin [German Health Survey: Use of complementary therapies].

[B13] Owen DK, Lewith G, Stephens CR (2001). Can doctors respond to patients' increasing interest in complementary and alternative medicine?. BMJ.

[B14] Rosemann T, Korner T, Wensing M, Gensichen J, Muth C, Joos S, Szecsenyi J (2005). Rationale, design and conduct of a comprehensive evaluation of a primary care based intervention to improve the quality of life of osteoarthritis patients. The PraxArt-project: a cluster randomized controlled trial [ISRCTN87252339]. BMC Public Health.

[B15] Altman R, Alarcon G, Appelrouth D, Bloch D, Borenstein D, Brandt K, Brown C, Cooke TD, Daniel W, Feldman D, . (1991). The American College of Rheumatology criteria for the classification and reporting of osteoarthritis of the hip. Arthritis Rheum.

[B16] (2000). Recommendations for the medical management of osteoarthritis of the hip and knee: 2000 update. American College of Rheumatology Subcommittee on Osteoarthritis Guidelines. Arthritis Rheum.

[B17] Katon W, Schulberg H (1992). Epidemiology of depression in primary care. Gen Hosp Psychiatry.

[B18] Kessler RC, Berglund P, Demler O, Jin R, Koretz D, Merikangas KR, Rush AJ, Walters EE, Wang PS (2003). The epidemiology of major depressive disorder: results from the National Comorbidity Survey Replication (NCS-R). JAMA.

[B19] Rosemann T, Backenstrass M, Joest K, Rosemann A, Szecsenyi J, Laux G (2007). Predictors of depression in a sample of 1,021 primary care patients with osteoarthritis. Arthritis Rheum.

[B20] Dickens C, McGowan L, Clark-Carter D, Creed F (2002). Depression in rheumatoid arthritis: a systematic review of the literature with meta-analysis. Psychosom Med.

[B21] Lowe B, Kroenke K, Herzog W, Grafe K (2004). Measuring depression outcome with a brief self-report instrument: sensitivity to change of the Patient Health Questionnaire (PHQ-9). J Affect Disord.

[B22] Lowe B, Spitzer RL, Grafe K, Kroenke K, Quenter A, Zipfel S, Buchholz C, Witte S, Herzog W (2004). Comparative validity of three screening questionnaires for DSM-IV depressive disorders and physicians' diagnoses. J Affect Disord.

[B23] Rosemann T, Korner T, Wensing M, Schneider A, Szecsenyi J (2005). Evaluation and cultural adaptation of a German version of the AIMS2-SF questionnaire (German AIMS2-SF). Rheumatology (Oxford).

[B24] Guillemin F, Coste J, Pouchot J, Ghezail M, Bregeon C, Sany J (1997). The AIMS2-SF: a short form of the Arthritis Impact Measurement Scales 2. French Quality of Life in Rheumatology Group. Arthritis Rheum.

[B25] McCullagh P NJA (1989). Generalized Linear Model.

[B26] Olfson M, Marcus SC, Druss B, Alan PH, Weissman MM (2003). Parental depression, child mental health problems, and health care utilization. Med Care.

[B27] Rosemann T, Joos S, Koerner T, Szecsenyi J, Laux G (2006). Comparison of AIMS2-SF, WOMAC, x-ray and a global physician assessment in order to approach quality of life in patients suffering from osteoarthritis. BMC Musculoskelet Disord.

[B28] Memel DS, Kirwan JR, Sharp DJ, Hehir M (2000). General practitioners miss disability and anxiety as well as depression in their patients with osteoarthritis. Br J Gen Pract.

[B29] Peat G, Thomas E, Handy J, Croft P (2004). Social networks and pain interference with daily activities in middle and old age. Pain.

[B30] Cronan TA, Hay M, Groessl E, Bigatti S, Gallagher R, Tomita M (1998). The effects of social support and education on health care costs after three years. Arthritis Care Res.

[B31] Thumboo J, Chew LH, Lewin-Koh SC (2002). Socioeconomic and psychosocial factors influence pain or physical function in Asian patients with knee or hip osteoarthritis. Ann Rheum Dis.

[B32] Creamer P, Hochberg MC (1998). The relationship between psychosocial variables and pain reporting in osteoarthritis of the knee. Arthritis Care Res.

[B33] Keefe FJ, Blumenthal J, Baucom D, Affleck G, Waugh R, Caldwell DS, Beaupre P, Kashikar-Zuck S, Wright K, Egert J, Lefebvre J (2004). Effects of spouse-assisted coping skills training and exercise training in patients with osteoarthritic knee pain: a randomized controlled study. Pain.

[B34] Dawson J, Linsell L, Zondervan K, Rose P, Carr A, Randall T, Fitzpatrick R (2005). Impact of persistent hip or knee pain on overall health status in elderly people: a longitudinal population study. Arthritis Rheum.

[B35] Hartel U, Volger E (2004). [Use and acceptance of classical natural and alternative medicine in Germany--findings of a representative population-based survey]. Forsch Komplementarmed Klass Naturheilkd.

[B36] Rao JK, Kroenke K, Mihaliak KA, Grambow SC, Weinberger M (2003). Rheumatology patients' use of complementary therapies: results from a one-year longitudinal study. Arthritis Rheum.

[B37] Artus M, Croft P, Lewis M (2007). The use of CAM and conventional treatments among primary care consulters with chronic musculoskeletal pain. BMC Fam Pract.

[B38] Joos S, Musselmann B, Miksch A, Rosemann T, Szecsenyi J (2007). GPs attitudes on complementary and alternative medicine (CAM) and its role within the German healthcare system - a focus group study. BMC Health Serv Res.

[B39] Grol R, Wensing M, Mainz J, Ferreira P, Hearnshaw H, Hjortdahl P, Olesen F, Ribacke M, Spenser T, Szecsenyi J (1999). Patients' priorities with respect to general practice care: an international comparison. European Task Force on Patient Evaluations of General Practice (EUROPEP). Fam Pract.

